# Perioperative Anxiety in Adults: A Narrative Review of Pathophysiology, Assessment, and Multimodal Management Strategies

**DOI:** 10.3390/healthcare14111561

**Published:** 2026-06-03

**Authors:** Jiashu Chen, Yuchi Zhuang, Meng Mao, Qinjun Chu, Zhengyuan Xia, Yan Wang

**Affiliations:** 1 Department of Anesthesiology and Perioperative Medicine, Zhengzhou Central Hospital Affiliated to Zhengzhou University, Zhengzhou 450007, China; chenchiashu@gs.zzu.edu.cn (J.C.); zyc020317@gs.zzu.edu.cn (Y.Z.); hnmaomeng@zzu.edu.cn (M.M.); mzk123456@zzu.edu.cn (Q.C.); 2 Department of Anesthesiology and Perioperative Medicine, The Second Affiliated Hospital of Zhengzhou University, Zhengzhou 450014, China; 3 Institute of Trauma and Metabolism, Tianjian Laboratory of Advanced Biomedical Sciences, Academy of Medical Sciences, Zhengzhou University, Zhengzhou 450052, China; 4 Cell Research and Transformation Center, Zhengzhou Central Hospital Affiliated to Zhengzhou University, Zhengzhou 450007, China

**Keywords:** perioperative anxiety, pathophysiology, assessment tools, biomarkers, intervention strategies

## Abstract

Perioperative anxiety is a common psychophysiological stress response experienced by patients before and after surgery, with a global prevalence of approximately 48%. Its occurrence is influenced by multiple factors including age, sex, type of surgery, and psychosocial determinants. The underlying pathophysiological mechanisms are complex, involving multi-system interactions such as autonomic nervous system imbalance, dysregulation of the hypothalamic–pituitary–adrenal (HPA) axis, dysfunction of limbic system neural circuits, and neuroinflammation. Current assessment strategies are evolving from sole reliance on psychological scales toward multimodal approaches incorporating objective biomarkers including heart rate variability, cortisol, and electroencephalography. Management paradigms have shifted from traditional pharmacological premedication to integrated systems encompassing structured patient education, digital health tools, neuromodulation techniques, and cognitive behavioral therapy. However, significant gaps persist regarding standardized screening protocols, biomarker validation, and targeted intervention pathways for high-risk populations. Future management is likely to require more individualized risk assessment and intervention selection. Biomarker-based risk prediction, artificial intelligence-assisted intervention decision-making, and the deep integration of digital therapeutics such as virtual reality with existing enhanced recovery pathways will be key directions for improving patient outcomes and recovery quality. This structured narrative review summarizes current evidence on perioperative anxiety in adults, focusing on epidemiology, pathophysiological mechanisms, assessment tools, biomarkers, and multimodal management strategies.

## 1. Introduction

Perioperative anxiety is a stress response experienced by patients facing surgery and anesthesia, often triggered by fear of the unknown, negative anticipation of pain and complications, concerns about loss of autonomy, and uncertainty regarding surgical outcomes. It frequently manifests as tension, fear, irritability, anger, and helplessness, and may even lead to catastrophic thinking [1]. Beyond being a distressing emotional experience, perioperative anxiety increases the risk of intraoperative adverse events such as arrhythmias and hemodynamic instability [2], and is associated with exacerbated postoperative pain, increased incidence of postoperative nausea and vomiting, elevated infection risk, prolonged hospital stay, as well as the occurrence of postoperative neurocognitive disorders and long-term chronic pain [3,4,5]. Therefore, accurate identification and effective management of perioperative anxiety are crucial for improving overall patient prognosis.

Unlike generalized anxiety disorder, perioperative anxiety typically presents as a “situational, phase-specific” response induced by the anticipation of surgery or anesthesia [6,7]. In recent years, with the widespread implementation of Enhanced Recovery After Surgery (ERAS) pathways and increased attention to anxiety in preoperative assessment, the management model for perioperative anxiety has shifted from simple reassurance and pharmacological premedication towards a comprehensive “assessment–education–intervention” system encompassing diverse strategies such as structured patient education, early psychological support, and optimization of pain and sleep [8,9]. However, whether to incorporate routine perioperative anxiety screening into preoperative protocols remains a subject of debate, with implementation often depending on the resources and workflows of individual healthcare institutions. Therefore, this narrative review summarizes the epidemiology, pathophysiological mechanisms, assessment approaches, and multimodal management strategies for perioperative anxiety in adults. By critically evaluating current evidence and identifying research gaps, we intend to inform clinical assessment, intervention selection, and future perioperative research.

## 2. Literature Search and Review Approach

A targeted literature search was performed in PubMed, Web of Science, and Embase. The main search period covered publications from January 2006 to 2026, and the search was last updated in April 2026. Earlier articles were included when they were widely cited, introduced commonly used assessment instruments, or provided foundational concepts relevant to stress physiology, anxiety neurobiology, or perioperative care. Search terms included combinations of “perioperative anxiety,” “preoperative anxiety,” “surgical anxiety,” “stress response,” “HPA axis,” “autonomic nervous system,” “neuroinflammation,” “assessment scales,” “biomarkers,” “heart rate variability,” “cortisol,” “electroencephalography,” “pharmacological intervention,” “non-pharmacological intervention,” “digital health,” “virtual reality,” “neuromodulation,” and “enhanced recovery after surgery.” Reference lists of key reviews and clinical studies were also checked to identify additional relevant sources.

The initial search retrieved approximately 450 records. Records clearly unrelated to adult perioperative care, perioperative anxiety, assessment, mechanisms, or management were not pursued further. Articles most relevant to the planned sections of the review were read in abstract or full text, and approximately 106 publications were retained as core references. Preference was given to studies in adult perioperative populations, randomized controlled trials, prospective cohort studies, systematic reviews, meta-analyses, clinical guidelines or consensus documents, and high-quality narrative reviews. Literature from general anxiety, fear, stress, or affective neuroscience was used only when perioperative-specific mechanistic evidence was limited; in such cases, the text identifies these interpretations as extrapolative frameworks rather than established perioperative mechanisms.

## 3. Epidemiology and Risk Factors

### 3.1. Global Prevalence

The reported prevalence of perioperative anxiety varies widely across surgical populations. A global meta-analysis estimated a pooled prevalence of preoperative anxiety of approximately 48% among surgical patients [10]. In low- and middle-income countries, the pooled prevalence was higher, reaching 55.7% [11]. This difference may be related to inconsistent preoperative counseling, limited access to anesthesia- and postoperative pain-related information, longer waiting periods, lower health literacy, and unmet information needs before surgery [11,12].Large observational studies from different healthcare systems have reported variable estimates. A cross-sectional study from the UK involving over 15,000 non-obstetric surgical patients revealed that despite a high patient satisfaction rate of 95%, 35% of patients still experienced perioperative anxiety [13]. In a German APAIS-based cohort of adult elective surgical patients, 40.5% had high preoperative anxiety, while the original APAIS validation study classified 32% of patients as anxiety cases [7]. In China, a national multicenter study of adult elective surgical patients using PAS-7 reported a prevalence of 15.8% [14], whereas another multicenter study using STAI-S greater than 44 reported high preoperative anxiety in 25.9% of patients [15].

Assessment tools also contribute to heterogeneity. In one comparative study, preoperative anxiety was detected in 46.4% of patients by APAIS, 44.4% by STAI, and 49.3% by ASSQ [16]. These instruments differ in focus: APAIS measures surgery- and anesthesia-related anxiety and information need; STAI-S measures situational state anxiety; ASSQ focuses on anxiety specific to surgery; and PAS-7 includes both psychological and somatic anxiety symptoms in Chinese surgical patients [14,15,16,17]. Accordingly, reported prevalence is shaped by the scale, threshold, language version, timing of assessment, and clinical setting.

### 3.2. Risk Factors

Risk factors for perioperative anxiety include sex, age, psychological antecedents, surgery- and anesthesia-related factors, and educational level. Among demographic factors, Female sex and younger age are frequently associated with higher reported anxiety. In Chinese multicenter data, preoperative anxiety was reported in 19.7% of female patients and 10.4% of male patients; the youngest age group had a prevalence of 24.5%, whereas the oldest group had a prevalence of 10.2% [14]. Meta-analytic data also support higher anxiety among female surgical patients, the observed sex difference may reflect not only biological susceptibility, but also differences in symptom reporting, cultural expectations, communication style, type of surgery, and willingness to disclose emotional distress [10,11]. Age-related findings are less consistent. Younger patients are often more concerned about anesthesia safety, intraoperative awareness, postoperative pain, interruption of work or family roles, and long-term functional outcomes [15]. Older patients may report less fear, but anxiety in this group may be complicated by frailty, multimorbidity, poor sleep, cognitive vulnerability, and depressive symptoms [18].

Psychological antecedents, such as trait anxiety, depressive tendencies, and previous negative surgical experiences, interact with environmental factors including surgical uncertainty, family separation, or comorbid psychiatric conditions, which can further exacerbate anxiety [19,20]. Surgical type and procedural risk are important clinical factors. Higher surgical risk has been associated with increased preoperative anxiety. In Chinese multicenter data, cardiac and obstetric/gynecological surgery showed the highest specialty-specific rates, both approximately 25%, whereas otorhinolaryngological surgery showed a lower rate of 10.3% [14]. Anesthesia-related concerns are also common. Patients undergoing highly invasive surgery most often reported concern about surgical outcome, followed by postoperative pain and anesthesia safety [15]. Other concerns include intraoperative awareness, anesthesiologist error, loss of control, not waking up from anesthesia, postoperative drowsiness, complications, and death [21,22,23,24]. Sleep disturbance is a modifiable factor. Preoperative anxiety was reported in 27.9% of patients with poor sleep and in 10.9% of those with good sleep [14]. Insomnia was independently associated with high preoperative anxiety, with an odds ratio of 1.79 [15]. Poor sleep may reflect preoperative worry, but it may also increase emotional reactivity, fatigue, and pain expectation before surgery. Furthermore, the impact of educational level is particularly pronounced in resource-limited settings, where lower educational attainment not only intensifies the experience of anxiety but also hinders its timely identification and effective management [12]. In a Palestinian surgical cohort, high information need was reported in 57.1% of patients, and high preoperative anxiety was reported in 27.1% [12]. Anesthesia counseling, procedure-specific education, sleep optimization, and pain planning are therefore practical targets for prevention. The major risk factors for perioperative anxiety are summarized in Table 1 by clinical domain, modifiability, and corresponding management implications.

## 4. Pathophysiological Mechanisms

The pathophysiology of perioperative anxiety involves complex interactions among the neuroendocrine, autonomic, and immune systems. These systems are activated by a combination of surgical stress, anesthesia, and psychological stressors, leading to pathological states such as persistent anxiety, cognitive bias, and abnormal cortisol levels or circadian rhythm disruption.

**Figure 1:**
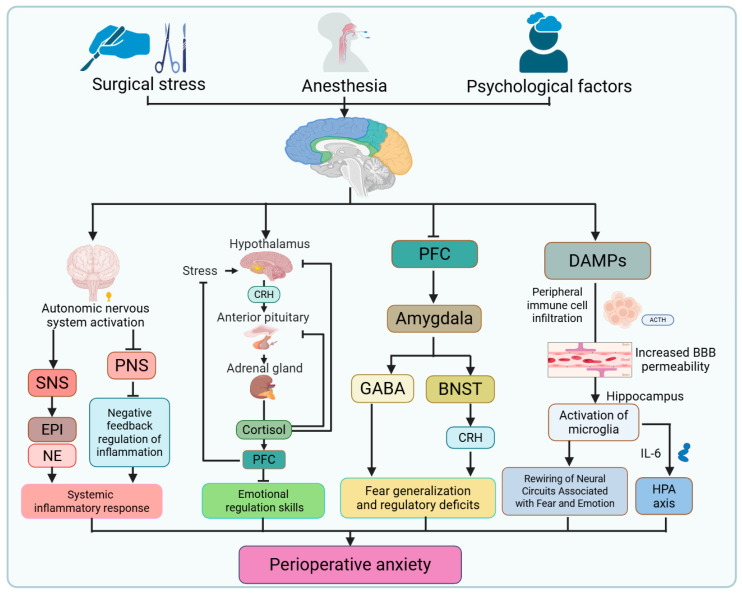
Pathophysiological mechanisms of perioperative anxiety. SNS: sympathetic nervous system; PNS: parasympathetic nervous system; EPI: epinephrine; NE: norepinephrine; CRH: corticotropin-releasing hormone; PFC: prefrontal cortex; GABA: γ-aminobutyric acid; BNST: bed nucleus of the stria terminalis; DAMPs: damage-associated molecular patterns; BBB: blood–brain barrier; IL-6: interleukin-6; HPA: hypothalamic–pituitary–adrenal. (→) indicates that an upstream component promotes a downstream target, (⊣) indicates that an upstream component inhibits a downstream target.

The mechanisms discussed in this section are supported by different levels of perioperative evidence. Autonomic arousal and HPA-axis activation have been directly examined in surgical patients using measures such as heart rate variability, anxiety scales, and salivary cortisol. Evidence related to prefrontal cortical activity is more limited but has begun to emerge from perioperative fNIRS studies. In contrast, several limbic and neurotransmitter mechanisms, including amygdala-centered threat processing, BNST-related anticipatory anxiety, and GABAergic or glutamatergic regulation, are mainly derived from broader anxiety and stress research. Figure 1 provides a schematic overview of the proposed pathophysiological mechanisms of perioperative anxiety.

### 4.1. Activation and Dysregulation of the Autonomic Nervous System

Perioperative anxiety is primarily mediated by the autonomic nervous system (ANS), characterized by activation of the sympathetic nervous system (SNS) and inhibition of the parasympathetic nervous system (PNS). SNS activation leads to the release of norepinephrine and epinephrine, triggering a cascade of responses including tachycardia, vasoconstriction, elevated blood pressure, and hyperglycemia. It also promotes a systemic inflammatory response, impairs immune function, and affects cardiovascular stability. A decrease in parasympathetic/vagal tone can lead to dysregulation of the inflammatory negative feedback loop, keeping the body in a prolonged state of high stress and further impairing emotional regulation and cognitive function [25,26,27]. Consequently, ANS imbalance is considered a crucial pathway linking psychological stress to multisystem physiological disturbances. Recent perioperative evidence supports this association. Guerrier et al. assessed patients before cataract surgery and reported significant associations between preoperative anxiety or surgical fear, measured using the Verbal Analogue Scale and Surgical Fear Questionnaire, and heart rate variability parameters. These findings suggest that HRV may reflect anxiety-related autonomic changes in the preoperative setting, although it should be interpreted as a non-specific physiological correlate rather than a standalone anxiety marker [28].

### 4.2. Regulation and Dysfunction of the Hypothalamic–Pituitary–Adrenal (HPA) Axis

The HPA axis is a major endocrine regulatory pathway involved in perioperative stress and anxiety. Psychological stress activates the hypothalamus to secrete corticotropin-releasing hormone (CRH), which stimulates the release of adrenocorticotropic hormone (ACTH) via the hypothalamic-pituitary portal system, leading to increased cortisol secretion from the adrenal cortex. In the short term, a moderate increase in cortisol helps enhance alertness, mobilize energy, and bolster cardiovascular responses to cope with impending surgical stress. However, when anxiety is excessive or persistent, it can result in sustained elevation of cortisol or disruption of its circadian rhythm [29]. Perioperative clinical evidence supports this stress axis involvement. In a prospective study of melanoma patients undergoing sentinel lymph node excision, Jansen et al. measured salivary cortisol together with HADS and STAI scores and observed an increase in salivary cortisol shortly before surgery. This finding supports the relevance of HPA axis activation in the perioperative setting, although cortisol should be interpreted as a non-specific stress marker rather than an anxiety-specific biomarker [30]. Chronically elevated cortisol levels can impair the cognitive control functions of the prefrontal cortex (PFC), predisposing patients to threat-related attentional bias and catastrophic thinking. Concurrently, disruptions in the sleep–wake cycle can further weaken the PFC’s capacity for emotional regulation, thereby establishing a vicious cycle of “anxiety–sleep disturbance–cortisol dysregulation” [31,32,33].

### 4.3. Limbic System Integration and Neurotransmitter Dysregulation

Perioperative anxiety arises from a synergistic dysregulation within limbic system neural circuits and their underlying molecular mechanisms. Preoperative stress, pain, or inflammation can impair the regulatory control of the medial prefrontal cortex (PFC) over the amygdala, leading to an aberrant enhancement of its output signals, which in turn precipitates and exacerbates anxiety [34,35]. The γ-aminobutyric acid (GABA)ergic inhibitory network within the amygdala acts as a critical “brake” limiting its over-excitation; a weakening of this function promotes the development and persistence of anxiety [36]. Within the extended amygdala, the bed nucleus of the stria terminalis (BNST) and its CRH-mediated connections are closely associated with sustained, anticipatory anxiety related to uncertain threats, facilitating the transition from acute stress to a chronic anxiety phenotype [37]. Evidence from broader anxiety and stress neuroscience studies suggests that PFC–amygdala connectivity is involved in emotional regulation, psychological resilience, fear generalization, and regulatory deficits [38,39]. Perioperative evidence at the prefrontal cortical level is also beginning to emerge. In an exploratory fNIRS study of patients undergoing functional endoscopic sinus surgery, Yang et al. reported that perioperative anxiety was associated with reduced PFC activation during a verbal fluency task, while computer-assisted cognitive behavioral therapy improved anxiety symptoms and enhanced PFC hemodynamic responses. These findings support the relevance of prefrontal regulatory function in perioperative anxiety, although they do not establish a complete amygdala–PFC circuit mechanism [40].

### 4.4. Neuroinflammation and Cytokine Interactions

Peripheral inflammation induced by surgical trauma can affect the central nervous system through multiple pathways, and may contribute to perioperative mood and cognitive disorders [41,42]. Damage-associated molecular patterns (DAMPs) and released cytokines activate pattern recognition receptors, altering blood–brain barrier permeability and promoting the infiltration of peripheral immune cells. This exacerbates microglial activation in the hippocampus and other brain regions involved in emotional regulation. Activated microglia release pro-inflammatory cytokines and mediate complement-dependent synaptic pruning, leading to the remodeling of neural circuits associated with fear and emotion [43,44]. Simultaneously, pro-inflammatory cytokines such as IL-6 activate the HPA axis, forming a positive feedback loop within the neuro-endocrine-immune axis, which may partly account for the association between persistent postoperative inflammation and long-term cognitive impairment [45,46].

## 5. Assessment and Diagnosis of Perioperative Anxiety

Accurate assessment of perioperative anxiety is fundamental for implementing targeted interventions, optimizing anesthetic management, and improving patient prognosis. Due to the highly subjective nature of the anxiety experience and its diverse manifestations, no single “gold standard” assessment tool currently exists in clinical practice. Available assessment methods can be broadly divided into subjective psychometric scales (Table 2) and objective biomarkers (Table 3).

### 5.1. Subjective Psychometric Scales

#### 5.1.1. State–Trait Anxiety Inventory (STAI)

The STAI consists of two 20-item subscales, the State Anxiety (STAI-S) and Trait Anxiety (STAI-T), each using a 4-point Likert scale with total scores ranging from 20 to 80 [47]. The STAI-S measures an individual’s transient anxiety level in a specific situation and is commonly used to establish a baseline for immediate preoperative anxiety, while the STAI-T reflects a person’s enduring predisposition to anxiety as a personality trait. The STAI requires a certain level of cognitive function from respondents and takes approximately 10–15 min to complete, which may limit its feasibility and accuracy in elderly patients, those with low educational levels, or in emergency/critical care settings. To enhance its practicality in clinical and research settings, Marteau and Bekker developed a brief six-item version of the STAI-S, which demonstrates a high correlation with the full scale and good internal consistency [48].

#### 5.1.2. Amsterdam Preoperative Anxiety and Information Scale (APAIS)

Developed by Moerman et al. in 1996 [17], the APAIS is a 6-item questionnaire: four items assess anxiety related to surgery and anesthesia, and two items assess the need for information. Each item is scored on a 1–5 Likert scale, and the average completion time is approximately 2–5 min, making it a suitable rapid screening tool for preoperative anesthesia visits or waiting areas. Clinically, a total score of ≥11 on the surgery/anesthesia-related anxiety items can serve as a criterion for identifying patients with high preoperative anxiety [49]. The strengths of the APAIS lie in its brevity and high specificity to the perioperative context; its limitation is the inability to assess personality or trait anxiety [17].

#### 5.1.3. Visual Analog Scale for Anxiety (VAS-A)

The VAS-A uses a continuous 0–10 (or 0–100) scale on which respondents mark their current anxiety intensity. It can be completed in seconds to one minute, making it suitable for rapid assessment and repeated measurements [50]. Studies have shown that the VAS-A has good sensitivity to short-term fluctuations in anxiety and exhibits moderate to high correlations with multidimensional anxiety scales [51]. However, as a unidimensional scale, the VAS-A cannot distinguish between the cognitive, emotional, and somatic dimensions of anxiety.

#### 5.1.4. Surgical Fear Questionnaire (SFQ)

The SFQ comprises eight items divided into two sub-dimensions: “fear of short-term consequences of surgery” (e.g., anesthesia, the operation itself, postoperative pain or complications) and “fear of long-term consequences” (e.g., health deterioration, delayed recovery, poor functional outcome). It is designed to systematically assess a patient’s fears and concerns regarding the “surgical/recovery outcome.” The SFQ can identify a patient’s specific sources of fear, thereby facilitating targeted preoperative education, postoperative rehabilitation, and psychological support [52].

Preoperative fear and preoperative anxiety overlap but should not be used interchangeably. Fear is usually directed toward a more specific object or consequence, such as postoperative pain, intraoperative awareness, anesthesia-related loss of control, surgical complications, or failure to recover. Anxiety is broader and may include diffuse worry, hypervigilance, sleep disturbance, somatic arousal, and a reduced sense of control before surgery [53]. This distinction has practical value. A patient with dominant surgical fear may benefit most from targeted information, anesthesia counseling, and pain planning, whereas a patient with high generalized anxiety, depressive symptoms, or trait anxiety may require broader psychological assessment and support [54]. Therefore, fear-specific tools such as the Surgical Fear Questionnaire complement, but do not replace, general anxiety or anxiety–depression screening tools.

#### 5.1.5. Hospital Anxiety and Depression Scale (HADS)

The HADS consists of two 7-item subscales: Anxiety (HADS-A) and Depression (HADS-D). It is a widely used screening tool for anxiety–depression comorbidity in clinical settings [55]. Its advantages include its brevity and good adjustment for confounding by somatic symptoms. However, it was not specifically designed for the perioperative setting and is often used clinically as a screening instrument for potential comorbid depression or a history of chronic anxiety. For patients with a positive HADS-A score, further evaluation using tools like the STAI or APAIS is recommended to guide intervention strategies [55].

#### 5.1.6. Perioperative Anxiety Scale-7 (PAS-7)

Developed and validated by Zhang et al. in 2021, the PAS-7 is a tool specifically designed for rapid perioperative anxiety screening [56]. It consists of seven items covering psychological and somatic dimensions and demonstrates good psychometric properties. Studies have shown that PAS-7 scores are significantly correlated with STAI-S and APAIS scores. Furthermore, in a Chinese patient population, the PAS-7 exhibited superior screening performance compared to the APAIS (PAS-7 AUC = 0.808 vs. APAIS AUC = 0.674) [57]. However, as the development and validation of the PAS-7 were based on a Chinese patient cohort, its applicability in other cultural contexts requires further investigation.

### 5.2. Objective Biomarkers

Although subjective psychometric scales may be influenced by recall bias, communication barriers, and assessor expectations, objective biomarkers for perioperative anxiety remain largely adjunctive and investigational. Current evidence suggests that physiological markers such as heart rate variability, cortisol, skin conductance, and EEG-derived indices may capture components of autonomic, endocrine, or cortical arousal. However, these markers are not specific to anxiety and may also be affected by pain, anesthetic exposure, surgical stress, inflammation, sleep disruption, medications, and comorbid disease. Therefore, they should not be interpreted as standalone diagnostic tools, but rather as complementary measures that may improve risk stratification when combined with validated clinical scales.

#### 5.2.1. Heart Rate Variability (HRV)

Heart rate variability (HRV) is a commonly used objective index for assessing autonomic nervous system function in the perioperative period. It includes time-domain indices such as the standard deviation of normal-to-normal RR intervals (SDNN) and the root mean square of successive RR interval differences (RMSSD), as well as frequency-domain indices such as low-frequency power (LF), high-frequency power (HF), detrended fluctuation analysis (DFAα1), and the LF/HF ratio [58]. These parameters reflect the balance between sympathetic and parasympathetic nervous systems. Preoperative reductions in HRV (e.g., decreased RMSSD/SDNN, elevated LF/HF) are closely associated with high anxiety levels and adverse postoperative outcomes including delirium, pain, and poor recovery [59,60]. HRV allows for objective, continuous monitoring of physiological stress and can be combined with subjective scales to enhance the predictive power for risk assessment. However, HRV measurements are susceptible to interference from factors such as environment, body position, and medications. Clinically, it is recommended to obtain a 5 min resting-state HRV measurement and integrate it with sleep and daytime dynamic assessments for a comprehensive evaluation of perioperative risk [61,62].

#### 5.2.2. Salivary or Plasma Cortisol

Cortisol is a core biomarker of the HPA axis stress response. Salivary cortisol, in particular, is a non-invasive measure sensitive to short-term psychophysiological stress and can be combined with subjective scales to establish a multimodal monitoring approach [63]. Elevated preoperative cortisol levels are positively correlated with anxiety scores and can predict postoperative cognitive function and recovery quality [64,65]. However, salivary cortisol is subject to significant diurnal variation and is influenced by factors such as diet, medication, and illness, necessitating standardized sampling and interpretation protocols [66].

#### 5.2.3. Skin Conductance/Electrodermal Activity (SC/EDA)

Skin conductance (SC) reflects the sympathetic nervous system’s regulation of peripheral sweat glands and is highly sensitive to emotional arousal and acute stress. Characteristic changes in fingertip electrodermal activity during painful stimuli or anesthetic induction allow for real-time, continuous monitoring [67,68]. Its advantages include rapid response and suitability for dynamic recording, making it applicable for capturing transient stress fluctuations during the perioperative period. However, SC is easily affected by ambient temperature and humidity, electrode placement, and non-specific sympathetic activation. Furthermore, a single SC value is difficult to quantify directly into an “anxiety level,” so it is primarily used clinically as a supplementary physiological monitoring indicator [67,69].

#### 5.2.4. Electroencephalography (EEG)

EEG provides real-time recording of brain oscillatory activity via scalp potentials and is a valuable tool for assessing perioperative brain function and emotional state. Anxiety states are often characterized by increased high-frequency beta power and decreased alpha power, reflecting prefrontal-limbic circuit imbalance and heightened cortical excitability [70]. Preoperative resting-state EEG metrics, such as alpha power, median frequency, and the prefrontal beta/alpha ratio, can predict the risk of postoperative attention deficits and delirium [71]. The strengths of EEG include its objectivity, non-invasiveness, and ability to provide real-time monitoring, making it particularly suitable for preoperative screening in high-risk elderly patients. However, it is sensitive to artifacts and lacks standardized thresholds. It is therefore recommended to integrate EEG with HRV and subjective scales in a multimodal prediction model, with careful attention to standardized data acquisition and analysis protocols [70,72]. Recent research suggests that machine learning-based multi-feature analysis of EEG can significantly enhance the prediction of anxiety and delirium, promoting the clinical translation of bedside EEG monitoring [73,74].

**Table 2:** Subjective Psychometric Scales for Perioperative Anxiety.

Scale Name	Most Suitable Clinical Use	Useful Patient Group or Setting	Main Value	References
Amsterdam Preoperative Anxiety and Information Scale (APAIS)	Rapid preoperative screening and identification of information needs	Preoperative clinic, anesthesia assessment area, patients with surgery- or anesthesia-related concerns	Brief tool that separates anesthesia-related anxiety, surgery-related anxiety, and need for information	[17,49]
State-Trait Anxiety Inventory (STAI)	Detailed anxiety assessment and research measurement	Clinical studies, trials, studies distinguishing state anxiety from trait anxiety	Well-established measure for state and trait anxiety; useful for baseline characterization and intervention studies	[47,48]
Visual Analog Scale for Anxiety (VAS-A)	Ultra-brief bedside or repeated anxiety rating	immediate pre-induction period, postoperative follow-up, mHealth monitoring	Very quick and easy to repeat; useful for tracking change over time	[50,51]
Surgical Fear Questionnaire (SFQ)	Assessment of surgery-specific fear	Patients whose main concern is pain, complications, recovery, or long-term surgical consequences	Captures fear of short-term and long-term consequences of surgery	[52,54]
Hospital Anxiety and Depression Scale (HADS)	Screening for anxiety–depression comorbidity	Hospitalized patients, older adults, patients with chronic disease or cancer, patients with suspected depressive symptoms	Assesses anxiety and depression while minimizing confounding from somatic illness symptoms	[55]
Perioperative Anxiety Scale-7 (PAS-7)	Perioperative-specific anxiety screening in Chinese adult surgical patients	Chinese perioperative populations, settings where somatic anxiety expression is clinically relevant	Includes mental and somatic anxiety symptoms; developed for adult surgical patients	[56,57]

**Table 3:** Objective Biomarkers for Perioperative Anxiety.

Biomarker	Key Metrics/Parameters	Clinical Significance and Application	Evidence/Appropriate Use	References
Heart Rate Variability (HRV)	Time-domain: SDNN, RMSSD; Frequency-domain: LF, HF, LF/HF ratio.	Reflects autonomic nervous system balance. Reduced preoperative HRV is associated with high anxiety and poorer postoperative outcomes.	Emerging adjunct. May be useful in research or perioperative monitoring when autonomic arousal is a major concern	[58,59,60,61,62]
Salivary/Plasma Cortisol	Salivary free cortisol concentration.	Reflects HPA axis activity. Preoperative elevation correlates positively with subjective anxiety and may predict postoperative cognitive function and recovery quality.	Emerging adjunct. Best used as an HPA axis stress marker in studies with standardized sampling	[63,64,65,66]
Skin Conductance (SC/EDA)	Skin conductance level and fluctuations.	Sensitive to acute emotional arousal and stress responses; suitable for real-time monitoring during key perioperative moments.	Emerging real-time arousal measure. Most suitable for short-term monitoring of acute sympathetic arousal in defined perioperative moments	[67,68,69]
Electroencephalography (EEG)	Spectral power (e.g., alpha, beta power; beta/alpha ratio), median frequency.	Anxiety states are often accompanied by increased beta and decreased alpha activity. Useful for assessing brain functional state and predicting postoperative delirium risk.	future stratification. More suitable for mechanistic studies and neurocognitive-risk research than routine anxiety screening	[70,71,72,73,74]

SDNN: standard deviation of normal RR intervals; RMSSD: the root mean square of successive RR interval differences; LF: low-frequency; HF: high-frequency; HPA: hypothalamic–pituitary–adrenal. Evidence maturity is based on the authors’ qualitative interpretation of the literature summarized in this narrative review and does not represent a formal GRADE assessment.

## 6. Treatment Strategies for Perioperative Anxiety

Interventions for perioperative anxiety differ substantially in evidence maturity, feasibility, mechanism, safety profile, and target population. Established approaches include structured perioperative communication, patient education, selected pharmacological anxiolysis, and psychological strategies adapted to the surgical context. Emerging adjuncts include VR/AR-based interventions, music therapy, aromatherapy, and non-invasive neuromodulation. Biomarker-guided and AI-assisted approaches remain future-oriented and investigational. Therefore, intervention selection should be guided not only by reported anxiolytic efficacy, but also by patient risk profile, safety, perioperative timing, feasibility, patient preference, and compatibility with institutional workflow. To facilitate clinical interpretation, the major interventions discussed in this section are compared in Table 4 according to evidence maturity, feasibility, target population, proposed mechanism, limitations, and suggested clinical role.

### 6.1. Pharmacological Interventions

Pharmacological interventions may be useful for selected patients with marked acute anxiety, but they should not be viewed as universally applicable first-line strategies. Their clinical value depends on the urgency of anxiolysis, need for sedation, patient age, respiratory risk, delirium risk, hemodynamic stability, and postoperative recovery goals.

#### 6.1.1. Benzodiazepines

Benzodiazepines are classic pharmacological agents for managing perioperative anxiety. They act by binding to the benzodiazepine site on the GABAA receptor, enhancing chloride channel activity, and inhibiting cortical and limbic systems, thereby attenuating HPA axis activation and reducing sympathetic tone [75]. Midazolam effectively reduces preoperative anxiety and decreases the requirement for anesthetic agents during induction. However, it carries a risk of respiratory depression, necessitating cautious use and enhanced monitoring in elderly, obese, or patients with respiratory compromise [76,77]. Remimazolam, an ultra-short-acting agent rapidly metabolized by esterases, offers controllable sedation, rapid recovery, hemodynamic stability, and a lower incidence of respiratory depression, making it particularly suitable for outpatient or ambulatory surgery [78].

#### 6.1.2. α2-Adrenergic Receptor Agonists

α2-Adrenergic receptor agonists, such as dexmedetomidine, activate central and peripheral α2 receptors, inhibiting norepinephrine release. This reduces sympathetic tone and enhances parasympathetic activity, producing sedative, analgesic, and stress-reducing effects [79]. A common clinical regimen involves an initial loading dose of 0.5–1 μg/kg infused intravenously, followed by a maintenance infusion of 0.2–0.7 μg/kg/h to achieve the desired depth of sedation. Studies indicate that compared to benzodiazepines, dexmedetomidine significantly improves sedation quality, alleviates anxiety, and reduces the risk of adverse postoperative outcomes [80,81]. However, dose-dependent bradycardia and hypotension require vigilance, and continuous hemodynamic monitoring is essential during its administration [79]. It should not, however, be described as a routine preoperative treatment for anxiety. Its use is most appropriate in monitored settings and in patients in whom the expected benefits of sedation and sympatholysis outweigh the risks.

#### 6.1.3. Gabapentin

Gabapentin has been studied as an adjunctive perioperative medication because of its effects on the α2δ subunit of voltage-gated calcium channels, reduction in excitatory neurotransmitter release, and potential modulation of pain sensitization. Some studies suggest that preoperative gabapentin may reduce short-term anxiety scores, particularly in highly anxious patients or in settings where anxiety coexists with anticipated postoperative pain. [82,83].However, its anxiolytic role should be interpreted cautiously. The potential benefits must be balanced against somnolence, dizziness, delayed recovery, oversedation, and possible additive respiratory depression when combined with opioids or other central nervous system depressants. These concerns are particularly relevant in older adults, patients with obstructive sleep apnea, frailty, renal impairment, or high postoperative opioid requirements. Therefore, gabapentin should be regarded as a selected adjunct for patients in whom anxiety and pain sensitization overlap [84,85].

#### 6.1.4. Ketamine

Ketamine, an NMDA receptor antagonist, exerts rapid antidepressant and anxiolytic effects by promoting synaptic plasticity and new synapse formation in prefrontal-hippocampal circuits [86]. Intravenous administration of low-dose ketamine (0.1–0.5 mg/kg) can produce a significant mood-improving effect within hours. However, this effect is typically transient and often requires repeated administration to sustain therapeutic efficacy [87,88].

#### 6.1.5. Melatonin

Melatonin can serve as a circadian rhythm regulator and a mild sedative–anxiolytic adjunct. Research indicates that preoperative oral administration of 3–10 mg of melatonin can reduce preoperative anxiety in adults, improve postoperative sleep quality, and decrease the incidence of delirium [89]. However, existing studies show considerable heterogeneity in dosing and timing, necessitating further standardized research to optimize its clinical application protocol.

### 6.2. Non-Pharmacological Interventions

#### 6.2.1. Perioperative Education

Management of perioperative anxiety should begin with identification of the patient’s dominant concern. For many patients, anxiety is driven by uncertainty, fear of anesthesia, anticipated pain, concern about surgical outcome, or lack of information rather than by a primary anxiety disorder. In these situations, structured preoperative information, individualized anesthesia counseling, expectation setting, and pain planning should be regarded as the foundation of care. Providing patients with clear, structured information about the surgery and perioperative processes can reduce uncertainty and fear of the unknown, promoting cognitive reappraisal to alleviate preoperative anxiety. Cognitive reappraisal involves the activation of prefrontal cognitive control networks and a concomitant downregulation of amygdala reactivity, constituting a plausible neural pathway from cognitive intervention to emotional attenuation [90]. Studies have demonstrated that multimedia or structured education is more effective than routine care in reducing preoperative anxiety [91]. This intervention is low-cost and easily integrated into routine perioperative workflows, but its effectiveness can be influenced by the patient’s educational level and information preferences.

#### 6.2.2. Digital Health Management

Digital health management includes augmented reality (AR) and virtual reality (VR). AR overlays interactive virtual guides onto the real environment, such as a “real-world walkthrough” of the surgical procedure and operating room before the day of surgery, which can reduce preoperative state anxiety levels more effectively than standard education [92]. VR offers a greater sense of immersion and can be used either as a short-term “distraction” intervention or as a desensitization tool centered on a virtual tour [93,94]. The advantages of digital health tools include rapid effectiveness from brief interventions, high reproducibility, the ability to personalize information and educational content, and the potential for integration with artificial intelligence (AI) systems for stratified intervention. However, their use is affected by equipment costs, disinfection protocols, sensitivity to motion, and varying patient acceptance of immersive experiences [95]. Furthermore, evidence for their long-term efficacy and underlying neuroimaging mechanisms remains limited, requiring further research using methods like functional MRI, along with multi-center large-sample trials and cost-effectiveness analyses, to support their clinical adoption.

#### 6.2.3. Transcranial Direct Current Stimulation (tDCS) and Transcutaneous Auricular Vagus Nerve Stimulation (taVNS)

tDCS and taVNS are emerging non-invasive neuromodulation techniques in the perioperative period. tDCS enhances the inhibition of limbic system activity by modulating PFC excitability, while taVNS activates vagal pathways to regulate autonomic balance and inhibit inflammatory mediators [96]. Clinical evidence shows that tDCS can effectively reduce the incidence of perioperative anxiety, and 15–30 min of taVNS can decrease subjective anxiety scores and improve HRV metrics [97,98]. However, standardization of stimulation parameters and the optimal timing for intervention remain unclear, warranting further clinical research for validation.

#### 6.2.4. Music Therapy

Music therapy can activate reward networks, including the nucleus accumbens and striatal-related circuits, modulate emotional reappraisal pathways, and alleviate anxiety by reducing sympathetic activation and downregulating HPA axis secretion. Studies have shown that music interventions can improve certain HRV indices and reduce stress-related biomarkers and preoperative subjective anxiety scores, although the effects are influenced by intervention duration, music genre, and the timing of measurement [99,100].

#### 6.2.5. Aromatherapy

Aromatherapy, often using essential oils like lavender (Lavandula angustifolia) administered via olfactory or local inhalation, can effectively reduce preoperative subjective anxiety scores [101]. In breast nodule biopsy procedures, inhalation of lavender or a lavender–peppermint combination has been shown to significantly reduce pain and anxiety [102]. Lavender’s constituents may exert calming and anxiolytic effects by modulating GABA receptors, influencing neurotransmitter systems, and affecting reward/limbic system activity. Aromatherapy can be accompanied by a decrease in cortisol levels or changes in autonomic nervous system function [103,104]. It offers advantages such as being non-invasive, highly acceptable to patients, and low-cost, although potential allergic reactions should be considered.

#### 6.2.6. Cognitive Behavioral Therapy (CBT)

Cognitive behavioral therapy (CBT) is a structured, problem-focused psychological intervention that works by identifying and modifying negative cognitive patterns and behavioral responses, thereby reducing catastrophic thinking, improving coping strategies, and enhancing self-efficacy. Compassion-based or emotion regulation-focused adaptations may further reduce HPA axis and sympathetic activation by influencing socio-emotional regulatory pathways and the prefrontal-limbic network [105]. Studies have shown that various CBT formats, including in-person preoperative sessions, brief interventions, or digital programs, can reduce preoperative anxiety and postoperative stress responses, as well as shorten hospital or ICU length of stay [106,107].

**Figure 2:**
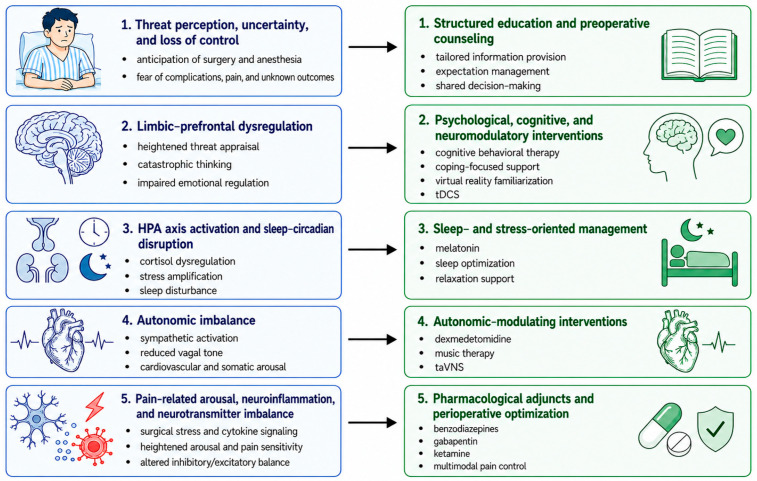
Integrated framework linking mechanisms of perioperative anxiety with targeted interventions. HPA: hypothalamic–pituitary–adrenal; tDCS: transcranial direct current stimulation; taVNS: transcutaneous auricular vagus nerve stimulation.

The effectiveness of perioperative anxiety management interventions lies in their ability to influence patients’ psychological and physiological states through multiple pathways, thereby promoting postoperative recovery. Future research should further elucidate the mechanisms underlying these interventions to optimize their application in clinical practice (Figure 2).

**Table 4:** Comparative appraisal of major interventions for perioperative anxiety in adults.

Intervention	Evidence	Feasibility	Target Population	Proposed Mechanism	Main limitations/Cautions	References
Structured education	Moderate–high	High	Most elective surgical patients; information-related anxiety	Reduces uncertainty; improves perceived control and coping	Depends on timing, content, health literacy	[88,89]
Benzodiazepines	Moderate–high	High	Short-term severe anxiety; rapid anxiolysis needed	GABAergic inhibition; reduced cortical-limbic excitability	Respiratory depression, delirium, delayed recovery	[75,76,77,78,79]
Dexmedetomidine	Moderate	Moderate	Sympathetic overactivity; need for sedation plus opioid-sparing	Sympatholysis; enhanced parasympathetic tone	Bradycardia, hypotension, monitoring required	[79,80,81]
Gabapentin	Low–moderate	Moderate	Anxiety with pain sensitization/high postoperative pain risk	α2δ calcium channel modulation; reduced excitatory neurotransmission	Somnolence, dizziness, oversedation, respiratory risk with opioids	[82,83,84,85]
Ketamine	Preliminary	Low–moderate	Selected affective symptoms or research settings	NMDA antagonism; synaptic plasticity	Psychotomimetic effects; limited anxiety-specific perioperative data	[86,87,88]
Melatonin	Moderate	High	Mild anxiety with sleep/circadian disturbance	Circadian regulation; mild sedative–anxiolytic effect	Heterogeneous dosing/timing	[89]
CBT/adapted psychological therapy	Moderate	Moderate	Catastrophic thinking, high trait anxiety, maladaptive beliefs	Cognitive restructuring; improved coping	Requires trained staff and time	[105,106,107]
Music therapy	Moderate	High	Patients preferring non-drug support	Emotional regulation; reduced sympathetic arousal	Variable music type, timing, duration	[99,100]
Aromatherapy	Low–moderate	High	Mild anxiety; comfort-oriented care	Olfactory–limbic modulation; relaxation response	Allergy, scent intolerance, formulation variability	[101,102,103,104]
Digital Health Management	Moderate/preliminary	Moderate	Moderate anxiety; procedural unfamiliarity	Distraction, exposure, reduced threat perception	Cost, cybersickness, infection control	[92,93,94,95]
tDCS/taVNS	Preliminary	Low–moderate	Selected high-anxiety patients/research settings	PFC modulation, vagal activation, autonomic regulation	Parameter heterogeneity, limited validation	[96,97,98]

tDCS: transcranial direct current stimulation; taVNS: transcutaneous auricular vagus nerve stimulation; Evidence is based on the authors’ qualitative interpretation of the literature summarized in this narrative review and does not represent a formal GRADE assessment.

## 7. Limitations and Future Perspectives

Several limitations should be acknowledged. First, although this review focuses on perioperative anxiety, not all proposed mechanisms are supported by the same level of perioperative evidence. Autonomic arousal and HPA axis activation have been examined directly in surgical patients using measures such as heart rate variability, anxiety scales, and salivary cortisol. In contrast, several limbic, neurotransmitter, and neuroimmune mechanisms remain less well characterized in perioperative populations. Concepts such as amygdala-centered threat processing, BNST-related anticipatory anxiety, GABAergic inhibition, and glutamatergic modulation are largely derived from broader anxiety and stress-neuroscience research. These mechanisms are relevant to perioperative symptoms, but they should not be interpreted as fully established mechanisms in surgical patients. Second, the existing clinical literature is heterogeneous. Reported prevalence varies according to surgical specialty, timing of assessment, healthcare setting, language and cultural context, anxiety scale, and cutoff value. Studies using STAI, APAIS, VAS-A, HADS, SFQ, or PAS-7 do not necessarily measure the same construct. Some tools capture general state anxiety, some focus on surgery- or anesthesia-related concerns, some assess fear of surgical consequences, and others are better suited for detecting anxiety–depression comorbidity. This heterogeneity limits direct comparison across studies and makes it difficult to define a universal prevalence estimate or a single screening threshold for all perioperative patients. Third, evidence for several newer interventions remains insufficient for routine implementation. Virtual reality, digital tools, tDCS, taVNS, aromatherapy, and biomarker- or AI-assisted stratification are promising but still limited by small samples, variable protocols, short follow-up, and inconsistent outcome definitions.

Future work should move from general anxiety reduction toward mechanism- and concern-based perioperative management. A practical patient-centered approach would begin with identifying the patient’s dominant concern: lack of information, fear of anesthesia, fear of pain, fear of complications, sleep disturbance, previous negative surgical experience, depressive symptoms, or high trait anxiety. Patients with information-related anxiety may benefit most from structured education, individualized anesthesia counseling, and expectation setting. Patients whose anxiety is driven by anticipated pain may need preoperative pain education and a clear postoperative analgesic plan. Patients with high trait anxiety, depressive symptoms, or catastrophic thinking may require psychological assessment or brief CBT-informed support. Patients with sleep disturbance may benefit from sleep optimization before surgery. This type of pathway is more clinically useful than applying a single intervention to all anxious patients.

Current perioperative anxiety management is advancing toward a “multimodal, patient-centered” approach. Future research and application should focus on promoting deep multidisciplinary integration, combining expertise from psychology, anesthesiology, surgery, and rehabilitation medicine to construct comprehensive management models. Concurrently, the rapid development of new technologies such as artificial intelligence, big data analytics, and telemedicine offers new possibilities for dynamic monitoring and precise intervention of perioperative anxiety. Integrating these cutting-edge technologies into clinical practice can not only enhance the sensitivity and specificity of anxiety identification but also enable real-time dynamic adjustments to individualized intervention plans, thereby significantly improving intervention efficacy and patient outcomes. At the methodological and implementation level, key innovative directions should include: (1) adopting a “patient–provider dyadic personalized continuous management” model, co-created from the patient’s perspective, to enhance intervention acceptance and effectiveness; (2) utilizing longitudinal mixed-methods research incorporating functional MRI, inflammatory profiling, and HRV to elucidate the dynamic roles of HPA axis and autonomic nervous system imbalance in the perioperative anxiety-recovery trajectory; (3) conducting high-quality, multi-center randomized controlled trials to compare the effects of multimodal non-pharmacological protocols versus conventional pharmacological regimens on postoperative delirium, functional recovery, and long-term quality of life; and (4) developing AI-assisted clinical decision support guidelines based on evidence-based interventions, combined with robust cost-effectiveness data, to facilitate the integration of effective interventions into standardized perioperative pathways such as ERAS.

## 8. Conclusions

Perioperative anxiety is a common psychological stress response in surgical patients and is associated with clinically relevant perioperative outcomes. Current research has made notable progress in assessment tools and intervention strategies, with a variety of scales and psychological approaches providing substantial support for clinical practice. However, discrepancies exist across studies regarding specific assessment metrics for anxiety, intervention timing, and the selection of methods. This highlights the need for more individualized and precise strategies in perioperative anxiety management. Future management goals should not only focus on alleviating anxiety symptoms but also strive to improve the patient’s overall surgical experience and quality of recovery. By optimizing anxiety management, improving the patient’s psychological state, enhancing postoperative pain control, reducing complications, and shortening hospital stays, the ultimate goal is the rational utilization of healthcare resources and the improvement of patients’ quality of life.

## Figures and Tables

**Table 1:** Contextual determinants and risk factors for perioperative anxiety.

Domain	Risk Factor	Modifiability	Clinical Implication	References
Demographic	Female sex	Non-modifiable	May indicate higher reported anxiety, but should be interpreted cautiously because sex differences may reflect biological, cultural, behavioral, and reporting-related factors	[10,11,14]
Demographic	Older age/frailty	Partially modifiable	Screen for anxiety–depression comorbidity, cognitive vulnerability, sleep disturbance, and delirium risk	[18]
Psychological	Trait anxiety, depression, catastrophizing	Partially modifiable	Consider structured psychological support, CBT-informed strategies, or referral	[19,20]
Surgical	High-risk surgery, cancer surgery, cardiac surgery, obstetric/gynecological surgery	Non-modifiable/procedure-related	Provide earlier information, expectation management, pain planning, and postoperative support	[14]
Anesthesia	Fear of loss of control, awareness, emergence, airway management	Modifiable	Targeted anesthesia counseling may reduce uncertainty	[21,22,23,24]
Social	Low health literacy, limited family support, economic stress	Partially modifiable	Use simplified education, teach-back, family involvement, and culturally adapted materials	[11,12]

## Data Availability

No new data were created or analyzed in this study. Data sharing is not applicable to this article.
